# Psychosocial and mental health challenges faced by emerging adults living with HIV and support systems aiding their positive coping: a qualitative study from the Kenyan coast

**DOI:** 10.1186/s12889-021-12440-x

**Published:** 2022-01-12

**Authors:** Moses K. Nyongesa, Carophine Nasambu, Rachael Mapenzi, Hans M. Koot, Pim Cuijpers, Charles R. J. C. Newton, Amina Abubakar

**Affiliations:** 1 KEMRI-Wellcome Trust Research Programme, Centre for Geographic Medicine Research (Coast), KEMRI, Box 230, Kilifi, Kenya; 2 Department of Clinical, Neuro- and Developmental Psychology, Amsterdam Public Health Research Institute, Vrije Universiteit Amsterdam, Amsterdam, Netherlands; 3 Department of Public Health, Pwani University, Kilifi, Kenya; 4 Department of Psychiatry, University of Oxford, Oxford, UK; 5 Institute for Human Development, Aga Khan University, Nairobi, Kenya

**Keywords:** HIV infections, Young people, Mental health, Psychosocial issues, In-depth interviews, Kenya

## Abstract

**Background:**

In sub-Saharan Africa, there is little data on the challenges faced by young people living with HIV transitioning into adult life. Adapting the socio-ecological framework, this qualitative study investigated the challenges faced by emerging adults living with HIV from a rural Kenyan setting. Additionally, the study explored support systems that aid positive coping among these young adults.

**Methods:**

In April 2018, in-depth interviews were conducted with a convenience sample of 22 young adults living with HIV (12 females), 18–24 years old, from rural Kilifi, coast of Kenya. Data were analyzed thematically using NVIVO 11 software.

**Results:**

Young adults living with HIV from this setting face various challenges at different levels of the social ecosystem. At the individual level, key challenges they reported included acceptance of HIV positive status, antiretroviral adherence, economic burden associated with access to healthcare, building an intimate relationship, mental health problems, and HIV status disclosure. At the family level, death of parents, poverty, and being unaccepted were the commonly mentioned challenges. At the community level, socialization difficulties and long waiting time at the HIV clinic were highlighted. HIV stigma and discrimination were frequently reported across the different levels. Economic independence, social support (from families, friends, organizations, healthcare providers and peer meetings), and reliance on spirituality aided positive coping among these young adults amidst the challenges of living with HIV.

**Conclusions:**

In this rural setting, emerging adults living with HIV face various challenges at the individual, family, and community level, some of which are cross-cutting. Our findings underscore the need for designing multi-level youth-friendly interventions that can address modifiable challenges encountered by emerging adults living with HIV in this and similar settings. Such interventions should incorporate appropriate context-specific support structures that may help these young people smoothly transit into adult life.

**Supplementary Information:**

The online version contains supplementary material available at 10.1186/s12889-021-12440-x.

## Background

Adolescence and young adulthood are crucial stages in the development of young people, a term we use here to combine the overlapping age groups of adolescents (10 to 19 years), youths (15–24 years) and young adults (18–24 years) [1, 2]. Millions of young people in these different age groups are living with HIV. As of 2019, 1.7 million young people aged 10 to 19 years were living with HIV globally [3], whereas 3.9 million young people aged 15 to 24 years lived with HIV worldwide by 2014 [4]. The majority of the young people living with HIV (YLWH) currently reside in sub-Saharan Africa (SSA) [5, 6]. The number of YLWH continues to grow globally because of an increased life expectancy of perinatally HIV-infected children with access to antiretroviral therapy (ART) and the high rates of HIV new infections among young people [3].

The challenges of living with HIV are numerous and include both psychosocial and socioeconomic stressors related to HIV and its care [7, 8]. At a young age, coping with HIV is even more challenging considering the expected life trajectory (schooling, working, marriage and parenthood) *vis-à-vis* the realities and setbacks of living with HIV. From SSA, there have been several qualitative investigations of the challenges particularly faced by YLWH [9–14], most reporting the views of younger adolescents 10 to19 years. Challenges commonly reported by YLWH from these studies include acceptance of HIV positive status, disclosure concerns, ART adherence, stigma and/or discrimination, parental loss, and chronic poverty. While it is true that some of these challenges are also shared by emerging adults living with HIV (18–24 years), this age group may be facing unique challenges specifically related to their developmental stage and further compounded by the situation of living with HIV. Young adulthood is a period of continued brain development especially in the prefrontal cortex area, where neurons undergo ‘pruning’ (unused neuronal connections shed off) and myelination (insulation of nerve fibers by myelin) to improve efficiency in executive functioning, including abstract thinking, problem-solving, planning and self-regulation [15, 16]. At 18 years and above, these young people are considered adults (by law and society) and are tasked with establishing independence (also financially) and assuming responsibility [17]. As the social network expands (e.g., new peers with advancing schooling or at the work environment), young adulthood is also a time for broadening social skills, for instance, to nurture romantic/intimate relationships often expected to lead into a lifelong relationship. Thus far, we are not aware of any qualitative enquiry on the living experiences of emerging adults living with HIV from SSA.

Despite quantitative studies noting a high prevalence of mental health problems among YLWH [18], qualitative inquiries of mental health challenges among YLWH in SSA remain limited. From the only qualitative study we are aware of in SSA [9], mental health appeared to be a challenge faced by younger adolescents (12 to 17 years) living with HIV. Several positive influences aiding socio-emotional coping among YLWH have been reported in the literature, but again the views emerge from only younger adolescents. These include spirituality [12, 14], social support [12–14], ego defense mechanisms such as rationalization and positive thinking [12, 13], ART availability and its treatment confidence [12, 13].

Transitioning from childhood to adult life is characterized by significant ecological, psychological and psychosocial changes and can be more daunting to a young person by the addition of an HIV diagnosis [9, 19]. No published study from SSA has so far explored the challenges faced by young people in the older end of the age spectrum of YLWH- the emerging adults. Most of the existing reports from this setting focus on younger age groups of YLWH, mostly adolescents 10 to 19 years. Furthermore, qualitative inquiries of mental health as a challenge among YLWH in general remain scarce calling for additional research. To our knowledge, only three studies from SSA [12–14] have investigated the support systems that help YLWH cope with the challenges of living with HIV, but again, all of them gather the views of only the younger age-group (adolescents 10 to 19 years). An in-depth understanding of the challenges alongside the support structures aiding positive coping of emerging adults living with HIV is therefore paramount to informing intervention approaches.

Based on an adaptation of the socio-ecological framework [20, 21], this qualitative study from rural Kenya aimed to explore: i) the challenges faced by emerging adults aged 18 to 24 years living with HIV (at an individual, family and community level); and ii) support systems aiding their positive coping. The strength of the socio-ecological approach is its recognition that experiences of health and illness are often shaped by factors within and beyond the individual [20, 21]. In this study, we do not focus on challenges at the public policy level of the socio-ecological framework. At the interpersonal level, we only focus on the family whereas the different organizational structures like school, hospital, and workplace are considered within the community level.

## Methods

### Study setting

This qualitative study was conducted in Kilifi County – coastal Kenya, at the Centre for Geographic Medicine Research in April 2018. Kilifi County is largely a rural setting with an estimated population of 1.5 million people [22]. The majority of its residents live below the poverty line [23]. Subsistence farming and fishing are the main economic activities in this County. Prevalence of HIV among individuals aged 15 years or above in Kilifi County is estimated at 4.5%, which is slightly lower than the national average prevalence of 6% [24]. In this setting, many young adults aged 18–24 years are schooling (in secondary schools or tertiary institutions) and still live with their immediate family or a relative [25].

### Study participants

Participants were emerging adults living with HIV, 18–24 years old, attending services at an HIV specialized clinic, Kilifi County Hospital. These young adults were identified from records of a previous study conducted in this facility [26] and were conveniently selected for potential participation based on availability and willingness to participate after the initial phone contact. During the recruitment process, an effort was made to enhance geographical and gender representation from the list of potential participants. Information saturation following individual interviews [27] is what informed a stop in recruitment of the emerging adults living with HIV and this was after the 22nd interview. None of the young adults we reached out to during the initial phone contact declined to participate in the present study.

### Data collection procedures

Individual in-depth interviews lasting 30–45 min were conducted with each participant by MKN and RM in private rooms at the HIV specialized clinic of the Kilifi County Hospital. All the interviews were done in Kiswahili (the official national language) and were audio-recorded using digital recorders after seeking consent from all participating youths. We used a semi-structured interview guide (see Additional file 1) to collect the data. The interview guide was informed by a mini review of the literature on key issues affecting people living with HIV [7, 8] and by the socio-ecological model [20, 21]. Based on these, a range of topics were covered, including psychosocial challenges (e.g., poverty, stigma, discrimination, interaction with peers, HIV disclosure, ART adherence) [7, 9, 10, 12–14], psychological wellbeing [9, 18], intimate relationship challenges [14], and the socioeconomic burden associated with living with HIV [7, 9]. In the discussions, participants were often probed about the level of the social ecosystem (individual, family, or community level) in which the mentioned challenge occurred if this was not clear. We also enquired about support systems that aided positive coping for these young adults facing different challenges of living with HIV, again informed by topics identified from the literature [12–14, 28].

### Data analysis

The data were analyzed thematically [29] using both deductive (i.e., pre-identified themes based on the interview guide) and inductive (i.e., emergent themes from the data) approaches [30] with the assistance of NVIVO 11 software (QSR International [Americas] Inc.). The final transcripts uploaded into NVIVO for analysis were based on the audio-taped materials transcribed verbatim, translated into English, and reviewed for accuracy. The analysis involved: i) immersion in the transcriptions; ii) development of an initial coding framework based on the interview guide questions as well as new emerging codes; iii) coding; and iv) separate comparative analysis of the codes. In steps (i) and (ii), authors MKN and CN familiarized with three randomly selected transcripts and each did an independent coding. To ensure acceptable inter-coder reliability, a meeting was then held between the two coders to discuss the coding framework. Differences in coding by the two coders were resolved through mutual agreement. In steps (iii) and (iv), MKN and CN independently coded additional seven transcripts and another meeting was held after which MKN proceeded to code the rest of the transcripts as the coding pattern was almost similar. Any emergent codes outside the agreed coding framework was first discussed between the coders before updating the codebook.

## Results

### Participant characteristics

In the present study, analysis involved a total of 22 transcripts. Table 1 summarizes the basic demographic characteristics of the study participants.

**Table 1:** Participant demographic characteristics

Characteristic	Frequency (%)
**Sex**	
Male	10 (45%)
Female	12 (55%)
**Age**	
18–19 years	10 (45%)
20–24 years	12 (55%)
Mean	20.7 years
**Education level**	
None	1 (4%)
Primary	7 (32%)
Secondary	11 (50%)
Tertiary	3 (14%)
**Religion**	
Christian	18 (82%)
Muslim	4 (18%)
**Residence in Kilifi County**	
Peri-urban areas ^a^	11 (50%)
Rural areas	11 (50%)

Note. ^a^ - these areas were within Kilifi county

### Challenges faced by young people living with HIV

**Fig. 1:**
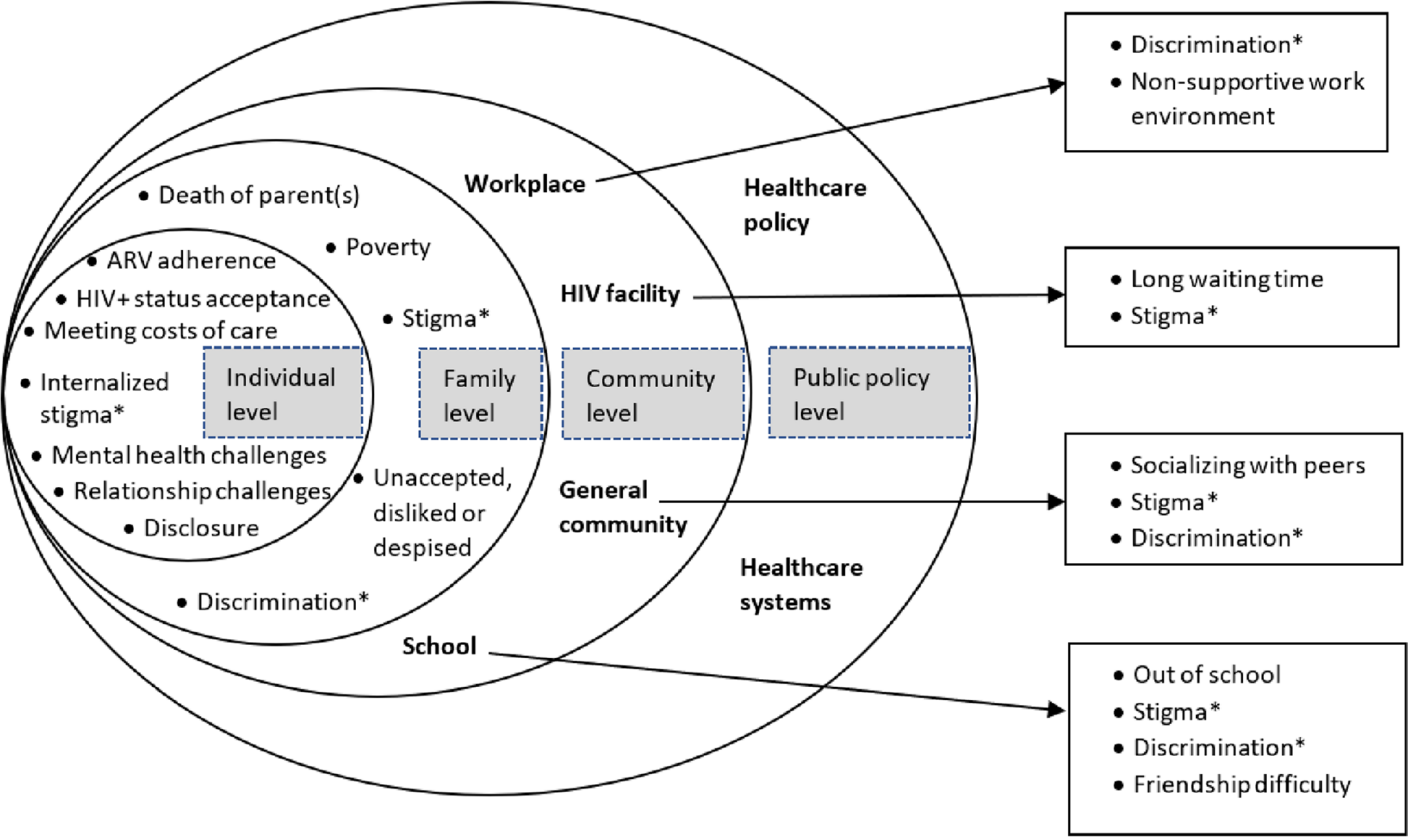
Challenges faced by young adults living with HIV from coastal Kenya, illustrated using a socio-ecological framework. In asterisk (*) are the inter-level challenges

Several themes emerged in our discussions with young adults living with HIV about the challenges they face in their day to day life at different levels of the socio-ecological system. Figure 1 provides a summary of these emergent challenges as themes. Below, we present each of the reported challenges in two ways. First, we present challenges reported from a specific level of the adapted socio-ecological system i.e. individual level challenges, challenges at the family level and community level (namely the general community, school, workplace, and the HIV facility). Second, we present inter-level challenges (those cutting across two or more levels). Illustrative quotes from each theme were selected for presentation through researcher consensus.

### Individual level challenges

#### Acceptance of HIV positive status

We asked the participants whether acceptance of HIV positive status was a challenge among young adults living with HIV. Many respondents discussed how it is difficult for some young people to come into terms with an HIV positive identity with some not wanting to be told they have HIV. Notably, none of the respondents directly reported this as being a challenge to self, instead talked about it as a challenge to ‘other’ YLWH. Nonacceptance of HIV positive status contributed to non-adherence to antiretroviral medication.

*There are some who will not accept and will say ‘I am this way now [living with HIV], it is better not to take those medications [antiretrovirals] and just wait to die’.*

*(Participant 12, Female, 18 years old).*

*S/he does not accept [HIV positive status] and does not want to be told they have HIV, now you get s/he no longer takes their medication [antiretrovirals].*

*(Participant 20, Female, 19 years old).*

A few participants however reported acceptance of HIV positive identity not being a challenge from the onset. To them, acceptance was like an eye-opener helping them to think of better ways of improving their life and health. It was easy to accept being HIV positive because of reasons such as being clearly informed of how one got the virus in the first place.

*For example within the community you come out and accept [being HIV positive], definitely you will have disclosed that status to several people and that also gives you the strength, you will say ‘now that I have accepted what next?’ Also, it gives your brain an allowance to think more, what can I do so that according to the state that I am in, yes, I have accepted, what will I do to sustain or improve my life, my health?*

*(Participant 7, Male, 19 years old)*

#### Disclosure of HIV positive status

Many of the participants discussed how disclosure was a challenge because of trust issues, fear of being asked too many questions (e.g. where they got the infection), being discriminated against, or the information spreading beyond those to whom they confide. Disclosing to people outside immediate caregiving family such as friends, relatives, romantic partners, or teachers (for those in school), was particularly difficult.

*That is a challenge that we also go through [disclosing HIV positive status], because it is my secret and I do not want you to know. Now you find if I tell this one, s/he will go tell others, when they speak out now others will start talking badly about me, and I will feel bad…and there are some who will even start discriminating [against] you.*

*(Participant 5, Female, 24 years old)*

Disclosure was mainly a challenge if one had not accepted her/his HIV positive identity.

*P: Haah! It is not a challenge [disclosing HIV positive status], once they have accepted their situation, things are easy.*

*I: What if they have not accepted?*

*P: Now there, s/he will face a great challenge.*

*(Participant 13, Female, 19 years old)*

#### Adherence to antiretroviral medication

This was a frequent challenge and was brought up by many of the interviewed young adults living with HIV from our setting. According to sentiments of one participant, which other participants also mentioned, young people in general have numerous activities of interest such as partying and travelling. In the process, those living with HIV may end up prioritizing such activities over the need to consistently take their antiretroviral medication.

*Young people particularly have a great challenge in adherence to drugs [antiretrovirals], because they have a lot of activities, unlike children and the elderly. There is lots of travelling, there is what they call partying etc, so taking medication can be a challenge because let us say on Friday, weekends most people like partying, you know? One [a YLWH] has a set time for taking her/his medication, like 8 or 9pm, then her/his friends come around 7pm and tell her/him ‘aaah, there is a party somewhere and we have fare, let’s go!’ That one will forget taking her/his medication because s/he has heard there is a party and s/he will not carry medication to take while at the party, so that is also a challenge.*

*(Participant 19, Female, 22 years old)*

Non-adherence to ART seemed to be a challenge particularly among young adults who had not accepted their HIV positive status. Other reasons that made it a challenge for young adults living with HIV to adhere to their medications included the size of the pills (being too big) and medication side effects such as dizziness.

*“It is really a challenge [antiretroviral adherence] for those who have not accepted living with HIV because someone will for sure refill their antiretroviral medication [at the HIV clinic]. Every time, they do not miss refilling, but now when it comes to taking the medication while at home, they don’t, you see?*

*(Participant 7, Male, 19 years old)*

*Now, others say the [antiretroviral] pill is big, it is difficult to swallow. Others say after taking the pills, they feel dizzy. Others say after taking the pills, they often feel hungry. These are some of the reasons why it becomes difficult for some to adhere.*

*(Participant 10, Female, 22 years old)*

With antiretroviral medication, adherence is not only about taking the pills regularly, but also ensuring that the time between dosages is regular. Some of the young adults described how it was difficult maintaining regular routine time of taking their antiretroviral medications because of reasons such as visitation by friends whom they have not disclosed to or conflicting school schedule.

*Now, maybe you are required to take [antiretroviral medication] at 8 o’clock, that time you find maybe you are required to be in a certain place and you must be there at that time and you cannot carry your medication and have them with you in class.*

*(Participant 1, Female, 21 years old)*

From the in-depth discussion around medication adherence with the participants, aspects of ‘getting tired’ and ‘giving up’ on daily antiretroviral dosage emerged. One of the participants was keen to point out that most young people living with HIV would have preferred other modes of administration of antiretroviral drugs such as monthly injections (if made available) compared to use of daily oral pills.

*You know most young people [living with HIV] say at least there should have been antiretroviral drug injections that one can use for a month after which they go back for another injection, at least that would have been better.*

*(Participant 9, Female, 24 years old)*

#### Intimate relationship challenges

Several participants discussed about the challenges of starting or sustaining an intimate relationship as a young adult living with HIV. From the discussions, it emerged that having a boy/girlfriend when in this age bracket is expected. However, many of the young adults do not disclose their HIV positive status to their relationship partners because of fear that such a relationship may end, and they will be heartbroken. For those whose status was known, or willingly disclosed their HIV positive status to a potential relationship partner, being rejected was not uncommon as explained in the following excerpt:

*You find that in the community I have friends, both male and female friends. Before I came to know about my [HIV positive] status, I had not started dating girls. Afterwards, I came to know about my status, and as I continued taking these drugs [antiretrovirals] and my viral load improved, I now wanted to be in a relationship with a girl. So, the very first time, the girl I talked to rejected me because I told her my [HIV positive] status, as I did not want to hide it from her. I told her the truth about being HIV positive and that I did not want to have a relationship with her so that I infect her, no, it was simply starting a close relationship, so we live happily in the community.*

*(Participant 14, Male, 19 years old)*

For some who were thinking about marriage, finding someone to start a lifelong relationship seemed a challenge, especially if that other person was HIV-uninfected. Others were uncertain about marriage life altogether, worrying for instance about how they will get married when they have HIV.

*Now, when you are in a relationship and you are this way [HIV positive], you question yourself a lot. The aim of starting a relationship is getting to know each other very well, because this often leads to marriage, and remember when you enter marriage life there is a time you will want to have children. So, a young person will question herself a lot, saying ‘this state of mine [being HIV positive], how will I get married? Will I give birth to children? Maybe the children will also be HIV positive.’ You understand?*

*(Participant 19, Female, 22 years old)*

#### Meeting costs of care

Many young adults talked of how meeting the costs of seeking HIV-related care was a personal challenge, especially the regular transportation costs to and from the HIV clinics for antiretroviral medication refill. Some described how they are forced to do hard menial jobs (mostly at the construction sites) to meet the regular costs of care.

*You are forced to do menial jobs at the construction site, in peoples’ farms, which is not an easy job, but because you have no alternative, you do it to meet the regular costs of care.*

*(Participant 6, Male, 22 years old )*

At worst, some have to go without taking their medication until when they can find the fare.

*Like me, I come from XXXX, you cannot walk from XXXX to Kilifi, it is far. So, you are forced to take a motorbike to the bus stop, at the bus stop you take a public service vehicle to here [Kilifi]. Now when you do not have fare, you are forced to stay at home even when your [antiretroviral] medication is finished, until when you get fare.*

*(Participant 20, Female, 19 years old)*

#### Mental health challenges

Common mental health problems such as depression and anxiety emerged as another challenge that young adults living with HIV face because of reasons such as losing parent(s) through HIV/AIDS, stigma and discrimination (explained in detail under the subsection on inter-level challenges), and external pressure to marry/get married when they perceived that their HIV situation may be a hindrance. These mental health problems were expressed by many of the participants we talked to often using idioms like “feeling sad”, “thinking too much”, and “worrying a lot” which are equivalent to local conceptualization of common mental health problems.

*Myself, I have a lot of sadness, I see others having their parents and I don’t, my parent died because of this HIV, so I am… [shaking head] until sometimes, it gets to a point, I cry.*

*(Participant 22, Male, 19 years old)*

*The way people say, aah! Young man, you need to marry. Now, because people do not know that I have this illness [HIV], I just keep quiet, then I go sit down and think too much, I have lots of thoughts, I say, aah! These people are telling me to marry and the way I am this way [living with HIV]?*

*(Participant 4, Male, 24 years old)*

Psychological distress can negatively impact the life of a young adult with HIV in that some may stop taking their antiretroviral medication, loose concentration in school (hence poor school performance) or think about committing suicide.

*When you are stressed up, you cannot even concentrate on other things. Maybe you are in class and stressed, you cannot keep up with studies. Also, if one [a YLWH] is stressed up, s/he cannot even do some things, s/he will think about many things, maybe regretting why it had to be like this [living with HIV] and what will happen in the long run. Now, such a situation can even discourage you, you decide even to stop taking the medication you were taking, you wait to die.*

*(Participant 11, Female, 20 years old)*

*When you are stressed or worrying a lot, there is another thing, you will say, ‘aah! Now why am I like this [living with HIV]? It is better I commit suicide, or I die through drug overdose’.*

*(Participant 18, Female, 22 years old)*

According to one participant, many affected young adults living with HIV end up abusing drugs as a coping strategy to avoid stressful thinking.

*Many end up using drugs of abuse, because of thinking a lot. They want to do away with such stressful thoughts, so they begin abusing drugs.*

*(Participant 3, Female, 21 years old)*

### Family level challenges

#### Parental loss

Most participants mentioned how loss of parent(s) can be challenging for a young adult living with HIV. A few participants specifically mentioned that they had lost one or both of their parents. Such parent(s) were a source of happiness to these young adults and played a key caretaking role including providing food. Parental loss greatly impacted on their lives. One participant mentioned getting stressed, losing weight and being unable to control their viral load (related to medication adherence problems) following parental death.

*The challenge that I have personally faced is when I lost my mother. Yeah, that affected me a lot, it was a great loss that I have never experienced in my life before, because she was my greatest source of support, she provided food, she stood by me, she was my happiness, but when she passed on, is when I started getting stressed until I lost weight, viral load went up to an extent I could not bring it down.*

*(Participant 2, Male, 22 years)*

Death of parents brought additional challenges to these young adults. Left on their own, some had to take the responsibility of meeting all their basic needs. Some had to move and stay with a relative or step-parent (where the remaining parent remarried), under whom the care was described by the young people as ‘not the same as before’.

#### Poverty

Poverty related issues including lack of food and financial problems within the family were described by many young adults living with HIV. Lack of food, for instance, made some young adults avoid taking their antiretroviral drugs (often strong and required to be taken after eating) while some reported taking their medication on an empty stomach. Financial challenges in the household made it difficult for a young adult living with HIV to have regular balanced diet and those depending on family support for transport/fare faced difficulty at the time they needed to go and refill their medication at the HIV clinic.

*Like me, I may sometimes not have money, no food, so you take medication [antiretrovirals] but you are hungry, you just take the medication like that [without eating].*

*(Participant 10, Female, 22 years old)*

*There is a challenge there, financial problems [in the family] contributes to a YLWH not having a balanced diet.*

*(Participant 6, Male, 22 years old)*

From the discussions with the young adults, it was clear that they are advised by healthcare providers to have at least five food servings in day but due to poverty, this is always not feasible. What most could manage was one food serving in a day (either in the morning or evening). Death of parents worsened the situation as they were often the primary providers of basic needs in the household.

#### Interpersonal relationship difficulty within the family

A few participants mentioned instances where themselves (or a young person they know living with HIV) were unaccepted, disliked or despised by immediate family members.

*Your brother, when he sees you taking [antiretroviral] medication, he starts despising you…saying, ‘you will remain that way, those drugs will not help; they even smell bad’*

*(Participant 17, Male, 18 years old)*

In some cases, this was because other siblings thought that they received ‘special treatment’ in the family.

*P: There is another challenge we go through. For a young person, but even any other person who lives with HIV, this virus does not go hand in hand with stress. So when your parent gets to know that you have this illness [HIV], and other siblings are well, there is a way they try beyond their means to cater for your needs first then those of other siblings, so that you do not become stressed and they ensure your health is good. These others [siblings] will begin saying you are being favoured.*

*I: Ehe, so when they say you are being favoured, in what way is that a challenge?*

*P: It can be a challenge because now all your siblings will be against you and they will not like you.*

*(Participant 19, Female, 22 years old)*

### Community level challenges

#### General community challenges

##### Socializing with fellow peers

Under this topic of discussion, young adults living with HIV were of divided opinion. Several of them talked about the difficulty in socializing with other peers within the community set-up because they feared that others will gossip about them, when or if they knew about their HIV positive status. This resulted in such young adults avoiding socializing with peers and living a solitary life.

*P: It is a challenge [peer-to-peer socialization] because you will see that when you are around your friends, you feel that they are talking badly about you, you feel uncomfortable.*

*I: Ehe, and how does that affect this young person?*

*P: Makes her/him not to socialize, s/he becomes lonely, always alone.*

*(Participant 16, Female, 24 years old)*

Other young adults living with HIV felt that socializing with peers was not a challenge if one had not disclosed their HIV status:

*Myself, I do not see any challenge, because you will not have told them that you have HIV or what, no. You will just be chatting with them and no one will be knowing. That will just remain your secret.*

*(Participant 10, Female, 22 years old)*

### Challenges within the school environment

#### Making close friendships

Only one participant raised this as a challenge. He described how difficult it was for a young person living with HIV to make close friendships within the school environment because of a fear of being suspected or asked detailed questions by such friends when seen taking medication routinely.

*You find that sometimes you fear making close friendships because you can…I mean when you get used to people, others can scrutinize you, for example at school, when you are taking [antiretroviral] medication on a daily basis, so they begin suspecting you and in that they can ask you a lot of questions, so this makes you fear a little bit. So…I mean such friendship becomes a problem sometimes.*

*(Participant 22, Male, 19 years old)*

#### Forced to be out of school

This was another challenge within the school environment as reported by one participant. In the wake of death of biological parents, relatives often take up caregiving roles but such care, as reported by the participant, may not compare to that from parents. A young adult living with HIV with a younger HIV-infected sibling may be forced to be out of school (sometimes for a very long time) to care for such a sibling when her/his condition deteriorates.

*For me, my parents passed away when I was very young. So, we were raised by our aunt, but the aunt was not raising us the way other children are raised. So, I could not go to school because I had my younger sibling whom this illness [HIV] was worsening. So, I had to stop schooling and studying completely and wait for my sibling to recover first before I continued.*

*(Participant 21, Male, 19 years old)*

### Challenges at work

#### Non-supportive work environment

According to one participant, some employers may not be understanding and supportive when it comes to the employee’s HIV-related health issues (in this case a young adult living with HIV) such as being allowed to go for the regular clinic appointments. This is despite the employer being aware of the HIV positive status of the employee. All they prioritize is their work being done.

*P: Another challenge is if one is employed, there is a way the boss may fail to understand. You will try explaining to her/him, but s/he will not want to understand. What s/he wants is for you to do her/his work but matters of your health is none of her/his concern.*

*I: Okay, is that after you have already told her/him about your HIV status, or you simply told her/him that you would like to go to the hospital?*

*P: Yeah, after you have already told her/him your HIV status.*

*(Participant 1, Female, 21 years old)*

### Challenges at the HIV facility

#### Long waiting time

The HIV clinic where we recruited the study participants is a high-volume facility attending to about 60 to 100 patients per day. A few participants raised long waiting time at the facility as a challenge mainly stemming from few health care providers who are available to offer services. Total time spent at the HIV clinic could be up to half a day.

*The service here needs to be improved a bit but also this [antiretroviral] medication need to be added [quantity] because for some of us who come from far, we come here [at the HIV clinic] we wait until 1pm is when we get our medication then we leave, now we suffer from hunger, we left home early morning only to come and wait here, the doctor is only one so we are told wait…and wait.*

*(Participant 15, Male, 18 years old)*

*This service would have been better because we can come here, here at the [HIV] clinic, you get few doctors, either one or two, so the services are very slow but when doctors are many, if we come here, we will be served quickly, one who needs to return to school goes, one who needs to go back to work goes.*

*(Participant 20, Female, 19 years old)*

### Inter-level challenges

Discrimination against young adults living with HIV and an experience of different forms of HIV-related stigma (internalized, perceived, enacted and associative) [9, 31] were mentioned by different study participants at multiple levels of the socio-ecological system. HIV-related discrimination was specifically mentioned at the family and community levels (general community, at school and when seeking employment). HIV-related stigma was experienced at an individual, family, and community level (general community, at school and at the HIV clinic). Table 2 presents the different forms of HIV-related discrimination and stigma experienced by these young adults. Select supporting quotes are also provided.

**Table 2:** Forms of HIV-related stigma and discrimination experienced by young adults living with HIV from coastal Kenya, according to the different levels of the socio-ecological system

Level of social ecosystem	Forms of HIV discrimination	Data sources	Select supportive quote(s)	Forms of HIV-related stigma	Data sources	Select supportive quote(s)
Individual level	–	–	–	▪Internalized stigma	4 young adults living with HIV	*Like me, when I come this side [Kilifi], I usually come to my sister, so I cannot take my medication [antiretrovirals] in front of her workers or other people at home, I usually hide so that I can take the medication.* *(Participant 21, Female, 24 years old)*
Family level	▪ Isolation ▪ Separate plate or cup to use ▪ Separate sleeping area ▪ Denied food ▪ Their education being stopped or changed to e.g. doing short courses ▪ Considered to be of no value ▪ Overwhelmed with chores unlike the rest of the children	13 young adults living with HIV	*Like me, I have a big problem. At home there is discrimination, I am given my own plate and told to sit aside alone, I am told ‘sit there you are worthless’. So, I am very sad at home, I am there but not happy. It is just that I don’t have an option but to stay, you wish to move out and go stay with your dad, but again it is very far.* *(Participant 15, Male, 18 years old)* *A person like me, when they tell me to wait, all of us have performed well, but my sibling has been taken to [secondary] school, not me. When they tell me to wait, I will start having a lot of thoughts. I will think maybe they are thinking when they educate me or when they pay my fees it is like wasting their money because it is like I will die any time.* *(Participant 19, Female, 22 years old)*	▪Perceived stigma ▪Enacted stigma ▪ Associative stigma	3 young adults living with HIV	*…they can say this person is HIV-infected, now when s/he mingles with my children, will s/he infect them? You realize in this they lack an understanding.* *(Participant 1, Female, 21 years old)* *Most will often be afraid sitting next to you or even sharing a bed with you, some house chores that need sharing like washing utensils, cooking, they will not want you to cook for them.* *(Participant 4, Male, 24 years old)* *Now I was not born with HIV, I got it after being born. Now I come to tell my parents, I am this way [living with HIV], it will take a lot of time for them to understand me. Now they can even chase me, telling me ‘go and stay on your own, fend for yourself because that [acquiring HIV] was your making’.* *(Participant 5, Female, 24 years old)*
Community level						
i. General community	▪ Being sidelined in community activities ▪ Friends keeping away once their HIV status is known	4 young adults living with HIV	*Now there is a way if one is living with this virus [HIV], now in the community there is this spreading of rumours, and then some people really lack knowledge about this virus. They think that when one gets the virus then they are not of any value to the community. You find maybe there is an activity that needs all young people to be involved maybe it is of benefit to them, but now that people in the community know that you are HIV positive, they sideline you.* *(Participant 19, Female, 22 years old)* *For young people like us [living with HIV], our life is usually very difficult. Even your friends can distance because XXXX now has HIV, you see?* *(Participant 9, Female, 24 years old)*	▪Enacted stigma	5 young adults living with HIV	*P: For me, one of the challenges I face, you may be going somewhere, and people there know that you have HIV, now when you pass, they begin saying ‘you see that lady, that lady has HIV. Now when it gets to that level, you begin thinking and questioning whether you have any value.* *I: Eee, what thoughts now?* *P: You can even think of committing suicide.* *I: Eee, have you ever planned to do so?* *P: Haah! Not yet, but such thoughts come and go* *(Participant 13, Female, 19 years old)* *Like me, I face a big challenge. I was very close with some friends in the community but then they were told, ‘aah! That boy, do not associate with him, when you greet him, he will infect you with HIV.’* *(Participant 15, Male, 18 years old)*
ii. At school	▪ A teacher being reluctant to mark work by a young person living with HIV ▪ Other students reluctant to play games with a young person living with HIV ▪ Other students reluctant to share anything with a young person living with HIV	3 young adults living with HIV	*You become discriminated in school, you are at school, maybe when you let the teacher know that on a certain day you will go to the clinic to refill medication, now s/he begins asking what the drugs are for, you are left with no option but to explain. Now when you tell her/him, s/he begins to discriminate you. Things to do with your books, s/he will…that is s/he will mark with caution or sometimes will never mark, that teacher will never mark that book. S/he will tell fellow students, the students also will discriminate you, they will not want to play [games] with you or share anything with you.* *(Participant 20, Female, 19 years old)*	▪Enacted stigma	2 young adults living with HIV	*At school there is also a challenge because from 18 to 24 years, most are in school, and students at times have problems, they are stubborn and intolerant. Now there [at school], when you are known to have HIV, first your desk-mate can run away when told ‘you are sitting next to an HIV-infected person, heeh! At your own risk, I cannot sit next to an HIV-infected person.’ Sometimes during breaktime, you may have bought foodstuff to share with friends. When others are given by their friends, they take, but when you want to share, they refuse ‘she has HIV, do not take her food, do not eat’, now those are the challenges.* *(Participant 19, Female, 22 years old)* *Another thing, maybe when other young people know that you have HIV, they will discriminate you or will not talk well about you. Friends will run away because they do not want to be in contact with you, maybe you can infect them.* *(Participant 11, Female, 20 years old)*
iii. At workplace (when seeking employment)	▪ Not considered for a job after health check ▪ Not considered for a job after disclosing HIV positive status	3 young adults living with HIV	*There are certain jobs where health tests are a must, and when one gets tested and is found to be HIV positive, s/he will not get the job* *(Participant 11, Female, 20 years old)* *Young people living with HIV, especially those who have completed secondary education or university or college, it is not easy for them to get a job so that they can sustain themselves. Wherever they go, when they decide to disclose that they are living with HIV, mostly, such young people are never considered and remain jobless.* *(Participant 21, Female, 24 years old)*	–	–	–
iv. At the HIV clinic	–	–	–	▪Perceived stigma	5 young adults living with HIV	*There is a big challenge here [HIV clinic], for example when a young adult like me coming from place XXXX, I am still in some form of denial, now you will say, ‘I do not want to go there [HIV clinic] and find so and so who will see me’, you see? Now you will have the fear that when you come here [HIV clinic] you will find the mother of so and so, ‘aah! So even the mother of so and so is here, she will go and tell other people’, you see? Now you give up [coming to the clinic]. Others will come in uniform [to be prioritized first], cover their faces with headscarf so that they are not recognized, others act as if they are staff at the clinic when they notice someone familiar, but actually they have come to refill their medication, you see?* *(Participant 3, Female, 21 years old)*

Note. - None mentioned

At the family level, HIV-related discrimination was more profound where a young person lived with a stepparent or a relative following the death or separation of biological parent(s).

*P: Most [YLWH] complain about discrimination.*

*I: Ehe, what do they say?*

*P: They say they are discriminated at home because they live with stepmother who has other children…they are given all the house chores, they are not valued, food is little when the other children are given enough, so it is a big problem.*

*(Participant 2, Male, 22 years old)*

In the community, HIV-related discrimination was characterized by rejection and isolation of YLWH by members of the community to an extent where already established friendships were terminated once one’s HIV positive status is known.

*The first challenge that many [YLWH] encounter is mostly discrimination; the society rejects them. For instance, a friend whom one used to share things with, when they come to realize their HIV positive status, from that day onwards, such friendship ends. (Participant 7, Male, 19 years old)*

From the in-depth interviews with young adults living with HIV, it emerged that in the school setting, discrimination from both teachers and fellow students arose from the knowledge about a young person’s HIV positive status (see select excerpt in Table 2). The prospects of discrimination made some young people not to disclose their HIV status. For those whose HIV positive status was known and had experienced discrimination, this negatively affected their ART adherence, concentration in class, and resulted into mental health issues.

*At school, when you are discriminated because of having HIV, you will not concentrate in class, you will be thinking a lot all the time asking, ‘why am I like this, why am I being discriminated?’*

*(Participant 18, Female, 22 years old)*

*You get stressed because you have no one to chat with [in the community], you are all alone.*

*(Participant 6, Male, 22 years old)*

Perceived and enacted forms of HIV-related stigma were the most commonly mentioned in our in-depth discussions with young adults living with HIV, at the family and community levels. Internalized (self) HIV-stigma was evident from the interview with one of the participants whereas associative HIV-stigma emerged in the discussions with another participant, at the family level (see Table 2 for select excerpts). At the community level, specifically the HIV clinics, HIV-stigma was more pronounced where a young person was still in denial of their HIV-positive status.

*When one has not accepted their [HIV-positive] status, it is very challenging to pass there [outpatient unit] and enter here [HIV clinic]. That is why sometimes you find a YLWH wearing something to cover their head, like a hijab, on the day of their clinic appointment.*

*(Participant 19, Female, 22 years old)*

Because of pervasive stigma in the community, it emerged that most young people opt for HIV services in clinics far away from their community.

*One will say, ‘aah! Why should I go there [a nearby HIV clinic]? There, people will see me and start talking. I’d rather come here [a faraway HIV clinic] where I am not known, although it is far.*

*(Participant 3, Female, 21 years old)*

For young adults who experienced stigmatization once their HIV-positive status became known, psychological distress, thoughts about committing suicide or running away from their community lingered on. Additionally, they struggled adhering to ART.

*Because of HIV-stigma, you find YLWH thinking about committing suicide, running away, and others are stressed.*

*(Participant 11, Female, 20 years old)*

### Support systems aiding positive coping among young adults living with HIV

**Fig. 2:**
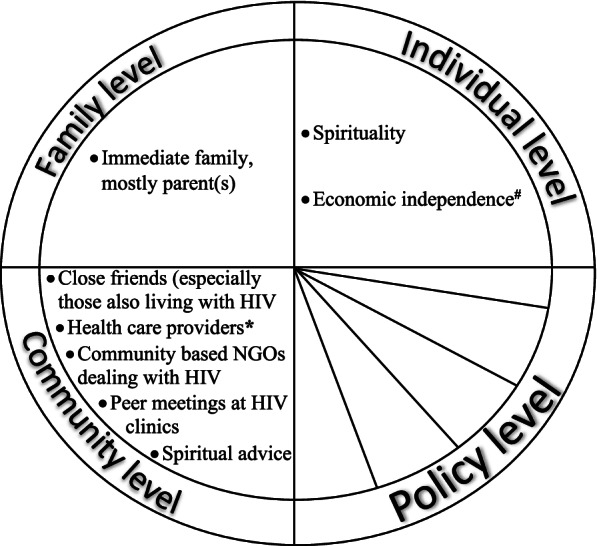
Support systems that aid positive coping for young adults facing different challenges of living with HIV in coastal Kenya, as illustrated using a socio-ecological framework. * includes clinicians, nurses, HIV counsellors and community health workers. ^#^ in the form of formal or self-employment. NGOs – nongovernmental organizations

We asked the participants about the pillars of support aiding their positive coping amidst the challenges of living with HIV. These young people mentioned several sources of support which we summarize in Fig. 2.

According to the views of several participants, economic independence (i.e., opportunity to work in formal or self-employment) not only keeps young adults living with HIV busy (therefore distracted from stressful thinking related to having HIV) but also the earnings thereof enables one to cater for basic needs including buying food and catering for own transportation needs during clinic appointment days.

*Us young people [living with HIV] do not have any other source of support other than a job. When you have a job, most of the stress is avoided because first, you will not be at home, maybe it is the home environment that is stressing. You will not be there; you will be busy involved with your development matters.*

*(Participant 7, Male, 19 years old)*

Reliance on spirituality, including advice from spiritual leaders, gives hope to young adults living with HIV (e.g. about emergence of a cure drug in the future) and guards against sexual promiscuity (which for instance may lead to acquisition of other HIV virulent strains).

*It can be a pillar because like a Christian, when you become religious, you cannot engage in some things out there, things like being promiscuous. You cannot do such things out there if you are religious.*

*(Participant 4, Male, 24 years old)*

The different forms of social support (from families, friends, community-based organizations, healthcare providers and peer meetings) described by the participants were, among others, a source of encouragement/moral support, financial support (mostly family and friends), advice and guidance (see Additional file 2 for more examples and illustrative quotes). As would be expected, healthcare providers play a key supportive role in terms of HIV treatment, care, and follow-up.

*They [doctors] are a source of support, a big pillar of support, because without them, without doctors, then it is death. Doctors are especially a strong pillar of support because when you arrive here [HIV clinic], when you explain yourself well, you get the best treatment.*

*(Participant 9, Female, 24 years old)*

## Discussion

### Summary of key findings

This qualitative study presents perspectives of emerging adults living with HIV from a rural setting in coastal Kenya regarding the challenges they encounter in day-to-day life and their pillars of support for positive coping. We utilized a socio-ecological model to explore these challenges. Young adults living with HIV expressed various challenges at the different levels of the social ecosystem. At an individual level, psychosocial challenges alongside emotional and socioeconomic challenges emerged as key concerns of living with HIV. At the family level, death of parents, poverty, and unacceptance were the frequent challenges whereas social interaction difficulties and long waiting time at the HIV clinic were the commonly mentioned challenges at the community level. Non-supportive work environment, being out of school and difficulty making friends at school were additional challenges mentioned by an individual than multiple participants. Notably, psychosocial issues of HIV-related stigma and discrimination were experienced by young adults living with HIV at multiple levels of the socio-ecological system. Being economically independent, social support and reliance on spirituality aided positive coping among the study participants amidst the challenges faced.

### Comparison of study findings with previous research

From previous research within SSA, psychosocial challenges like what we report have been documented not only among YLWH [8, 9, 11–14] but also the adult population living with HIV [7]. According to a recent systematic review of qualitative studies from East Africa [8], HIV-related stigma (in its various forms) and discrimination, ART nonadherence, HIV disclosure concerns, and struggling with an HIV-positive identity, are the most common of the psychosocial challenges faced by younger adolescents (12–19 years) living with HIV. In the current study, these also emerged as frequent challenges faced by young adults (18–24 years) living with HIV. Therefore, previously recommended interventions for addressing the psychosocial challenges faced by younger adolescents living with HIV [10, 13] can also be extended to address similar challenges in young adults living with HIV. These include skill-building interventions to prepare young adults for HIV self-management and HIV status disclosure, interventions building stronger positive self-concept in young adults living with HIV, beyond their HIV-positive status and intensified counselling services to improve their ART adherence.

The fact that HIV remains a stigmatized illness three decades later is a conundrum to policy implementers [9]. In this study, many young adults living with HIV mentioned an experience of different forms of HIV-related stigma and discrimination at multiple levels. HIV-stigma forced some young adults to seek care at HIV clinics distant away from where they lived. In part, this explains why many of the young adults living with HIV mentioned meeting the costs of HIV care, especially transport costs, a challenge. For other young adults, HIV-stigma and discrimination resulted into problems with ART adherence, schooling difficulties, and mental health concerns including psychological distress and suicidal thoughts. In the literature, HIV-stigma has been identified a strong predictor of both suicidal ideations and attempts among HIV patients in SSA [32]. Tackling HIV-related stigma and discrimination will require continued community sensitization on HIV/AIDs (including current advances) and where possible this should be integrated with other ongoing intervention programmes involving community members.

Psychosocial challenges of intimate relationship and financial difficulties especially in meeting the costs of HIV-related care appear unique to young adults living with HIV. These challenges are hardly mentioned in studies involving younger age groups of YLWH. In a study that recruited YLWH 12 to 24 years [14], intimate relationship challenges appeared a quandary. We think that this topic may have been brought up by older participants in the studied group – the emerging adults. For challenges that are unique to young adults living with HIV, a focused intervention approach is recommended. For instance, implementation of various youth-friendly sexual and reproductive health services [33, 34] using strategies that work in SSA [33] can be a good avenue for these young adults to discuss about their future life goals at an individual level. Where feasible, transport support programmes to young adults living with HIV is encouraged to promote treatment adherence leading to better HIV outcomes.

In the context of HIV, the relationship between some of the reported psychosocial issues is very complex and one psychosocial factor cannot be prioritized for intervention without a consideration for another. For instance, from our data, struggling to come into terms with HIV positive identity contributed to poor ART adherence and disclosure difficulties whereas anticipated HIV discrimination deterred young adults living with HIV from disclosing their HIV positive status, an observation also reported in the literature [13, 35]. Furthermore, psychosocial issues can lead to mental health problems in people living with HIV [14, 36]. Therefore, when designing interventions for addressing psychosocial challenges of YLWH in general, inter-relatedness of these factors should be considered.

This study is among the few studies with a qualitative design to investigate psychological challenges faced by YLWH in SSA and the first to involve emerging adults with HIV. Many participants described an experience of common mental health problems like depression and anxiety often using local idioms. According to the views of caregivers and service providers involved in a previous qualitative study conducted in this setting [9], younger adolescents living with HIV also experience psychological distress. Our finding further complements the quantitative observation that mental health problems are common among YLWH [5, 18]. Mental health problems like depression are particularly of concern since they have been associated with suicidal tendencies, even in YLWH [37]. In this study, suicidal ideations because of psychological distress also emerged from the discussions with several participants. More research, preferably of qualitative design, is needed to comprehensively understand mental health issues faced by YLWH in the larger SSA. Since mental health problems in the African setting are often expressed using local terms or idioms rather than the Western terminologies [38], local conceptualization of mental health and illness is paramount for any meaningful investigation.

We found that interpersonal relationship within the family setting was a challenge among young adults living with HIV where some reported being unaccepted, disliked or despised. This finding has hardly been reported in the literature making it difficult to compare. Vulnerability to rejection at the family level has however been reported by a South African qualitative study involving key populations living with HIV [7]. Another study from Iran reported unacceptance of people with HIV as a challenge but within the community set-up [39]. Other challenges that emerged at the family level in this study have previously been reported among YLWH, that is, loss of parents [13, 14] and poverty [9, 13]. Expressing grief for loss of a loved one is a normal bereavement process across cultures [13] but the problem arises when prolonged grieving negatively impacts on the life of the affected individual. For some of our participants, parental loss amplified psychological distress probably due to uncertainty of how the future will hold in the absence of their primary dependency. Continued counselling to help emerging adults living with HIV successfully come out of the grieving stages is encouraged.

In Africa where rates of poverty and unemployment are high [40, 41], it is not surprising that poverty and financial difficulties restricting access to medical care were brought up as a challenge in the discussions with young adults living with HIV. These socioeconomic issues have also been previously reported by other studies conducted in different African regions and involving YLWH [8, 9, 13, 14]. Where resources are constrained, the required adjustments for a healthy living post an HIV-positive diagnosis (such as need for balanced diet, transport during regular clinic visits for check-up and medication refill) can be additional economic burden to persons living with HIV or their family. In fact, a recent study from this setting [42] found a high burden of both direct and indirect costs among caregivers of YLWH, with the key drivers of direct costs being transportation and medication. Additional economic burden may lead to HIV-related negative outcomes. For instance, from our data, lack of enough food was among the reasons for poor ART adherence in the discussions with some young adults living with HIV whereas lack of fare contributed to missed medical appointments. Guarding persons living with HIV or their families against HIV-related economic burden or negative outcomes thereof will require interventions that look at HIV treatment beyond availing free ART such as household economic strengthening interventions.

Even though some young adults living with HIV did not consider social interaction with other peers in the community a challenge, especially when their HIV status was not known, others found this to be a concern. We are not aware of any reports on socialization difficulties among youthful population living with HIV to compare our findings, but a study conducted in Iran reported social frustration as a challenge faced by adult population living with HIV [39]. In this study, a few of the young adults living with HIV pointed out long waiting time at the HIV clinic (up to half a day) as a challenge they faced attributable to few healthcare providers. This challenge, alongside others like non-supportive work environment for these young adults and them being forced out of school to take care of ailing siblings are pointers of public policy (structural) level issues, requiring further research understanding. For example, there may be inadequate funding at the public policy level for deploying more doctors to the HIV facilities, therefore patients have to bear with the long waiting time. Extended waiting time at the HIV clinics is a barrier to HIV service utilisation [43] with a potential for contributing to the reported high clinic attrition among YLWH [44].

The factors aiding positive coping among young adults living with HIV reported in this study are congruent with what has been reported in prior research [10, 12–14, 28]. Social support from family and friends can play a key role in helping YLWH adhere to ART medication for instance by reminding them to take ART. Through social support, YLWH can also get guidance on healthy living. According to a previous review, young people with low levels of parental and family support are more likely to engage in sexual risk behaviours [45] hence higher risk of HIV acquisition or transmission. Support from social groupings can bolster self-esteem and create an environment where YLWH encourage and motivate each other. In a Ugandan study [46], access to support groups was predictive of higher levels of self-concept whereas in Zambia [10], youth groups provided friendship, encouragement and motivation to continue taking ART. The ability to make an earning through formal or self-employment made it possible for some study participants to fend for themselves without having to rely on family or other sources for support. This finding points to the need for inclusion of young adults living with HIV in youth economic empowerment programmes such as trainings on self-employment opportunities. This recognises that formal employment opportunities are limited and often require some level of specialized training and experience which a YLWH may not have. For young adults living with HIV with a strong religious background, their spiritual beliefs should be considered when offering care and advise, as appropriate.

### Study limitations

This study was conducted in a single geographical area in Kenya and findings may not necessarily be generalizable to other regions outside the study setting. Respondents were a convenience sample of emerging adults receiving HIV care and views expressed in this study may not be representative of all young adults living with HIV in the study area. Since we were interested in experiences of young adults living with HIV themselves, we only used a single qualitative data collection approach than multiple data sources which would have allowed for the triangulation of the information given. By using a guided semi-structured interview, we may have missed other relevant issues of concern to young adults living with HIV. We did not focus on challenges experienced at the public policy level. Future studies can take this line of inquiry. Lastly, explicit gender-based analyses of the challenges experienced by young adults living with HIV were beyond the scope of this work.

### Implications for intervention, research and care

The limitations above notwithstanding, this study has important implications for intervention, future research and care of emerging adults living with HIV as outlined below.

1. The challenges faced by young adults living with HIV at the different levels of the social ecosystem are multi-faceted. Addressing them will need design and implementation of multi-level interventions in this or other similar settings. Such interventions should be youth friendly (like these sexual and reproductive health programmes [33, 34]) and incorporate appropriate support systems for positive coping of these young adults.
2. The multi-faceted challenges experienced by young adults living with HIV in our setting may interfere with brain development processes at the prefrontal cortex. Therefore, it would be important for future studies to examine executive functioning skills and higher-order cognitive abilities at the developmental age of 18–24 years, in the context of HIV.
3. The emergent psychological challenges among these young adults living with HIV call for formal assessment of common mental health problems at the HIV clinics to allow early initiation of appropriate care for those found to have significant mental health issues. This is particularly important since poor clinical outcomes such as disease progression and poor ART adherence have been associated with common mental health problems co-occurring with HIV [47].
4. Preference for long acting antiretroviral agents over daily oral pills that emerged from the discussions with young adults living with HIV emphasizes the need to propel research on different long acting agents as additional options that may improve ART adherence among people living with HIV.
5. HIV is among one of the many chronic illnesses confronted by young people across the globe. Some of the challenges raised by young people in this and other previous studies could also be shared across other chronic illnesses. It would be interesting therefore to learn about the lived experiences of young people living with other chronic illnesses such as cancer, diabetes, sickle cell disease etc.
6. Long waiting time at the HIV clinics was mentioned as challenge by some participants in this study and this may contribute to the high attrition from care observed among YLWH [44]. Where possible, hospital management should seek to improve service delivery at the HIV clinics by for instance increasing service personnel or increasing the quantity of dispensed medication for young adults living with HIV so that they less frequently have to visit the facility unless otherwise.
7. As mentioned earlier, long waiting time at the HIV clinics alongside the challenges of young adults being forced out of school or encountering non-supportive work environment are pointers of public policy level challenges. An in-depth understanding of public policy level challenges will need a separate research investigation.
8. For young adults struggling to come into terms with HIV+ identity or those who have lost their parents, continued counselling should be offered to support them through to acceptance.

## Conclusions

This study shows that emerging adults living with HIV in our setting experience a range of challenges at an individual level but also at different levels of their ecological environment. Notably, the confronts of HIV-related stigma and discrimination were reported at multiple levels of the socio-ecological system. To fully address these challenges, most of which are modifiable, our findings underscore the need for designing and implementing multi-level, youth-friendly interventions in this and similar settings. Such interventions should incorporate appropriate context-specific support structures that may help these young people smoothly transit into adult life.

## Supplementary Information

**Additional file 1.** Interview guide for in-depth interviews with young adults living with HIV from coastal Kenya.

**Additional file 2.** Support systems that aid positive coping among young adults facing different challenges of living with HIV in coastal Kenya.

## Data Availability

No additional data available. The data that support the findings of this study have been included in this article (and its supplementary information files).

## Abbreviations

YLWH: Young people living with HIV
ART: Antiretroviral therapy
SSA: Sub-Saharan Africa

## References

[1] Patton GC (2016). Our future: a lancet commission on adolescent health and wellbeing. Lancet.

[2] WHO. Orientation Programme on Adolescent Health for Health-care Providers. 2006; Available from: https://www.who.int/maternal_child_adolescent/documents/pdfs/9241591269_op_handout.pdf.

[3] UNICEF. Adolescent HIV prevention. 2020; Available from: https://data.unicef.org/topic/hivaids/adolescents-young-people/.

[4] UNAIDS. Active involvement of young people is key to ending the AIDS epidemic by 2030. 2015; Available from: https://www.unaids.org/en/resources/presscentre/featurestories/2015/august/20150812_PACT.

[5] Evangeli M (2018). Mental health and substance use in HIV-infected adolescents. Curr Opin HIV AIDS.

[6] UNICEF. For Every Child, End AIDS: Seventh Stocktaking Report, 2016. 2016; Available from: https://www.unicef.org/publications/files/Children_and_AIDS_Seventh_Stocktaking_Report_2016_EN.pdf.pdf.

[7] Cloete A (2010). Challenges faced by people living with HIV/AIDS in Cape Town, South Africa: issues for group risk reduction interventions. AIDS Res Treatment.

[8] Kimera E (2019). Challenges and support for quality of life of youths living with HIV/AIDS in schools and larger community in East Africa: a systematic review. Syst Rev.

[9] Abubakar A (2016). ‘Everyone has a secret they keep close to their hearts’: challenges faced by adolescents living with HIV infection at the Kenyan coast. BMC Public Health.

[10] Denison JA (2015). “The sky is the limit”: adhering to antiretroviral therapy and HIV self-management from the perspectives of adolescents living with HIV and their adult caregivers. J Int AIDS Soc.

[11] Mburu G (2014). Responding to adolescents living with HIV in Zambia: a social–ecological approach. Child Youth Serv Rev.

[12] Mutumba M (2015). Psychosocial challenges and strategies for coping with HIV among adolescents in Uganda: a qualitative study. AIDS Patient Care STDs.

[13] Petersen I (2010). Psychosocial challenges and protective influences for socio-emotional coping of HIV+ adolescents in South Africa: a qualitative investigation. AIDS Care.

[14] Ramaiya MK (2016). A qualitative exploration of the mental health and psychosocial contexts of HIV-positive adolescents in Tanzania. PLoS One.

[15] Simpson, R., W. Kettyle. Young Adut Development: Brain Changes. 2018; Available from: https://hr.mit.edu/static/worklife/youngadult/brain.html.

[16] Teipel, K. Developmental Tasks and Attributes of Late Adolescence/Young Adulthood (Ages 18–24 years). n.d.; Available from: http://www.amchp.org/programsandtopics/AdolescentHealth/projects/Documents/SAHRC%20AYADevelopment%20LateAdolescentYoungAdulthood.pdf.

[17] Bonnie RJ, Stroud CE, Breiner HE (2015). Young adults in the 21st century, in Investing in the Health and Well-Being of Young Adults.

[18] Vreeman RC, McCoy BM, Lee S (2017). Mental health challenges among adolescents living with HIV. J Int AIDS Soc.

[19] Betancourt TS (2013). Annual research review: mental health and resilience in HIV/AIDS-affected children–a review of the literature and recommendations for future research. J Child Psychol Psychiatry.

[20] McLeroy KR (1988). An ecological perspective on health promotion programs. Health Educ Q.

[21] Stokols D (1996). Translating social ecological theory into guidelines for community health promotion. Am J Health Promot.

[22] KNBS. Kenya Population and Housing Census Volume I: Population by County and Sub-County. 2019; Available from: https://www.knbs.or.ke/?wpdmpro=2019-kenya-population-and-housing-census-volume-i-population-by-county-and-sub-county.

[23] CRA. Kenya County Fact sheets. 2011; Available from: http://siteresources.worldbank.org/INTAFRICA/Resources/257994-1335471959878/Kenya_County_Fact_Sheets_Dec2011.pdf.

[24] NACC. Kenya HIV County Profiles 2016; Available from: http://nacc.or.ke/wp-content/uploads/2016/12/Kenya-HIV-County-Profiles-2016.pdf.

[25] Nyongesa MK (2021). Prevalence, risk and protective indicators of common mental disorders among young people living with HIV compared to their uninfected peers from the Kenyan coast: a cross-sectional study. BMC Psychiatry.

[26] Nyongesa MK (2019). Prevalence and correlates of depressive symptoms among adults living with HIV in rural Kilifi, Kenya. BMC Psychiatry.

[27] Fusch PI, Ness LR (2015). Are we there yet? Data saturation in qualitative research. Qual Rep.

[28] Lypen KD (2015). “When we are together I feel at home.” Types and sources of social support among youth newly diagnosed with HIV in Kenya: implications for intervention. Afr J AIDS Res.

[29] Braun V, Clarke V (2006). Using thematic analysis in psychology. Qual Res Psychol.

[30] Azungah T (2018). Qualitative research: deductive and inductive approaches to data analysis. Qual Res J.

[31] Grappone G (2017). The seven types of stigma.

[32] Necho M, Tsehay M, Zenebe Y (2021). Suicidal ideation, attempt, and its associated factors among HIV/AIDS patients in Africa: a systematic review and meta-analysis study. Int J Ment Heal Syst.

[33] Obiezu-Umeh C (2021). Implementation strategies to enhance youth-friendly sexual and reproductive health Services in sub-Saharan Africa: a systematic review. Front Reprod Health.

[34] UNFPA. Adolescent sexual and reproductive health. 2014; Available from: https://www.unfpa.org/resources/adolescent-sexual-and-reproductive-health.

[35] Lyimo RA (2014). Stigma, disclosure, coping, and medication adherence among people living with HIV/AIDS in northern Tanzania. AIDS Patient Care STDs.

[36] Stutterheim SE (2009). HIV-related stigma and psychological distress: the harmful effects of specific stigma manifestations in various social settings. AIDS.

[37] Wonde M (2019). The magnitude of suicidal ideation, attempts and associated factors of HIV positive youth attending ART follow ups at St. Paul’s hospital Millennium Medical College and St. Peter’s specialized hospital, Addis Ababa, Ethiopia, 2018. PloS One.

[38] Bitta MA (2019). Priority mental, neurological and substance use disorders in rural Kenya: traditional health practitioners’ and primary health care workers’ perspectives. PLoS One.

[39] Dejman M (2015). Psychological, social, and familial problems of people living with HIV/AIDS in Iran: a qualitative study. Int J Prev Med.

[40] Mariama S (2017). Figures of the week: sub-Saharan Africa’s labor market in 2017.

[41] UNDP and OPHI. Global Multidimensional Poverty Index 2019: Illuminating Inequalities 2019; Available from: http://hdr.undp.org/sites/default/files/mpi_2019_publication.pdf.

[42] Katana PV (2020). Economic burden and mental health of primary caregivers of perinatally HIV infected adolescents from Kilifi, Kenya. BMC Public Health.

[43] Tafuma TA (2018). Barriers to HIV service utilisation by people living with HIV in two provinces of Zimbabwe: results from 2016 baseline assessment. South Afr J HIV Med.

[44] Lamb MR (2014). High attrition before and after ART initiation among youth (15–24 years of age) enrolled in HIV care. AIDS (London, England).

[45] Perrino T (2000). The role of families in adolescent HIV prevention: a review. Clin Child Fam Psychol Rev.

[46] Atwine B, Cantor-Graae E, Bajunirwe F (2005). Psychological distress among AIDS orphans in rural Uganda. Soc Sci Med.

[47] Kemigisha E (2019). Prevalence of depressive symptoms and associated factors among adolescents living with HIV/AIDS in South Western Uganda. AIDS Care.

