# Supplementary material for: Psychosocial and mental health challenges faced by emerging adults living with HIV and support systems aiding their positive coping: a qualitative study from the Kenyan coast
Source: BMC Public Health. 2022 Jan 12;22:76. doi: 10.1186/s12889-021-12440-x (PMC8756635; doi:10.1186/s12889-021-12440-x)
Supplement: Supplementary file 1 — Additional file 1. Interview guide for in-depth interviews with young adults living with HIV from coastal Kenya. [file 12889_2021_12440_MOESM1_ESM.pdf]

Additional file 1: Interview guide for in-depth interviews with young adults living with HIV from coastal Kenya

Instructions to the interviewer:
Before the interview begins, conduct the informed consent. Also, collect demographic data from the interviewee (sex, age, education level, religion, place of residence) and record in the provided register.
Introduction (~5 minutes)
- Welcome participant and introduce yourself.
- Explain the general purpose of the discussion (e.g. this discussion will be about the challenges faced by young people, 18-24 years old, living with HIV) and why the participant was chosen (e.g. because you are a young person in this age category and receiving HIV services at the KCH clinic).
- Discuss the process of the in-depth interview
- Explain further the presence and purpose of recording equipment
- Address the issue of confidentiality (e.g. that information discussed is going to be analyzed as a whole and that the participants' names will not be used in any analysis of the discussion).

Discussion Guidelines
NB: Probes will be used to ensure all important areas are covered

1. Challenges of living with HIV
There are specific challenges that people living with various chronic conditions such as HIV face. In your opinion, what challenges do you or your fellow peers living with HIV/AIDS face in day to day life? If not clear from the discussion, probe as follows…the challenge(s) you have mentioned, where does it occur? At HOME, SCHOOL, WORK, HIV CLINIC, in the general COMMUNITY or at an INDIVIDUAL level?

Anything else you would like to add?

Areas to Probe ...ONLY if not mentioned or described in detail from the above opening question.
- What about accepting HIV positive status, is it a challenge to YLWH?
- Socialization/interaction with peers?
- Stigma and discrimination?
- Accessing healthcare services/service friendliness (what are the barriers and facilitators?)
- Adherence to antiretroviral medication?
- Disclosure difficulties? (if raised ask…to whom and why it is easy/difficult to disclose or not to disclose)
- Other psychosocial challenges? e.g. poverty, financial challenges, loss of parent(s)/partner
- Psychological challenges? emotional problems such as anxiety, depression, and stress (or their local conceptualization)
- Economic burden arising from living with HIV/AIDs? E.g. medical expenses, hospital bills, transport or fare to/from the HIV clinic
- Challenges of sexual and intimate relationship?

2. Coping strategies

What are some of the sources of strength or support aiding better coping among young people faced with different challenges of living with HIV?

Areas to Probe (if not mentioned) …
- Does spirituality/religion have a role in helping young people cope with HIV?
- DO family/friends have a role?
- Healthcare systems? role of doctors, counsellors, community health workers, peer support groups

We have come to the end of this interview. Do you have anything else you would like to add? Do you have any questions?
