# Supplementary material for: Psychosocial and mental health challenges faced by emerging adults living with HIV and support systems aiding their positive coping: a qualitative study from the Kenyan coast
Source: BMC Public Health. 2022 Jan 12;22:76. doi: 10.1186/s12889-021-12440-x (PMC8756635; doi:10.1186/s12889-021-12440-x)
Supplement: Supplementary file 2 — Additional file 2. Support systems that aid positive coping among young adults facing different challenges of living with HIV in coastal Kenya. [file 12889_2021_12440_MOESM2_ESM.pdf]

Additional file 2: Support systems that aid positive coping among young adults facing different challenges of living with HIV in coastal Kenya

Support system	Data sources	Ways of support	Illustrative quote(s)
Family (mostly parents)	14 young adults living with HIV	- Offer encouragement/moral support<br>- Giving advice and guidance (especially if biological parents also live with HIV)<br>- Facilitation e.g. fare to the clinic<br>- Reminding a young person living with HIV about their clinic appointment date<br>- Reminding a young person living with HIV the time for taking their antiretroviral medication	*If you have your parents, it will be easy to get support because they will be advising you, alongside how doctors will be counselling you, they will also be advising you at home, how to use this medication [antiretroviral], eating well, and continuing normally with life (Participant 18, Female, 22 years old)*<br><br>*Family is a strong pillar of support, they help me a lot, the family is of help to us young people living with HIV that is. I am given fare to come here [HIV clinic], and for going back [home], and when they help me that way, when I also get money, I refund.*<br>*(Participant 15, Male, 18 years old)*
Close friends (especially those also living with HIV)	14 young adults living with HIV	- Offer encouragement/moral support<br>- Giving advice<br>- Reminding a young person living with HIV the time for taking their medication (especially at school setting)<br>- Chatting with a friend helps to keep off negative thoughts that affect mental wellbeing<br>- Financial support to buy basic needs e.g. food, soap and clothing<br>- Accommodate a young person living with HIV if unaccepted in their family.	*P: Friends are also a pillar of support*<br>*I: Eee, in what way?*<br>*P: Like myself, I used to think a lot and be lonely when I knew now I have the virus [HIV]. Now, as I routinely came for counselling, I got one friend here [HIV clinic]. Now, he was the one encouraging me saying 'aaah! Let this not worry you, this is not sickness.*<br>*(Participant 4, Male, 24 years old)*<br><br>*P: Like myself when at home and I feel I have a lot [of thoughts], I just go to my friends for a chat and that feeling goes away eventually.*<br>*(Participant 13, Female, 19 years old)*
Economic independence# (in the form of employment opportunities)	7 young adults living with HIV	- Enables a young person living with HIV buy food (one participant mentioned that availability of food improves adherence to medication)<br>- Enables a young person living with HIV cater for their transportation costs during clinic appointment days<br>- Keeps a young person living with HIV busy and engaged therefore lesser negative thoughts that affect mental wellbeing	*I: What else do young people rely on as their pillar of support in living with HIV?*<br>*P: Work.*<br>*I: Ehe, when you say work, what do you really mean?*<br>*P: If you are working you get an earning.*<br>*I: Ehe, and how will that help, or how is it supportive?*<br>*P: It is a pillar of support because if one has a job, s/he gets to earn a salary at the end of the month which can be used to cater for personal needs, transport to*

			go for counselling from the doctors or for medication [antiretroviral] refill.<br>*(Participant 4, Male, 24 years old)*
Spirituality and advice from spiritual leaders	13 young adults living with HIV	- Gives a young person living with HIV the confidence to open out<br>- Protects against sexual promiscuity (which can lead to acquisition of other HIV virulent strains)<br>- Gives hope and encouragement<br>- Spiritual leaders give advice and guidance	*When it comes to the spiritual aspect, for instance, when someone goes to let's say the church, there is…there are some books [Bible chapters] that offer encouragement making one know that 'aaah! I can also continue normally with life', other than just staying at home thinking too much about the situation one is undergoing.*<br>*(Participant 11, Female, 20 years old)*<br><br>*…like myself, I have an uncle who told me to pray to God, one day I will be healed. So, I usually pray knowing that one day God will help me in that there will be a cure drug.*<br>*(Participant 22, Male, 19 years old)*
Health care providers *	16 young adults living with HIV	- Counselling a young person living with HIV about the importance of medication adherence<br>- Encouraging a young person living with HIV to continue taking their medication<br>- Offering medical care<br>- Trusted for advice since they have known the young person living with HIV for long<br>- Supporting those who are out of care in the community back to receiving care (mainly the role of community health workers)	*I: Do you think young people living with HIV rely on health care providers including community health workers for support in coping with the challenges of living with HIV*<br>*P: Eee, they are of great support because others choose to remain at home, they do not want to honor clinic appointments, now when they are visited by community health workers, they return to care.*<br>*(Participant 10, Female, 22 years old)*
Peer-to-peer support meetings at the HIV clinics	2 young adults living with HIV	- Helps a young person living with HIV to open and talk about their problem<br>- Getting advice	*When they meet, that is the young people living with HIV, you find a person feels as if they have met a brother or sister, that is you find yourself in another family other than the one you live with, and the way they [other young people living with HIV] treat you is different. When we meet as young people living with HIV, you find we become like brothers and sisters, it is easier to open and talk about what is bothering you.*<br>*(Participant 1, Female, 21 years old)*
Nongovernmental organizations (NGOs) implementing HIV-related	1 young adult living with HIV	- Supporting people living with HIV including the youths	*P: The second source of support is that we have many organizations, and when the organizations are many, people living with HIV must be benefiting.*<br>*I: When you say many organizations, which are some of these?*

projects in the community			*P: Nongovernmental organizations such as NILINDE, AFYA PWANI, NAC INTERNATIONAL, there is PATHFINDERS etc…all these big organizations, there is KEMRI, so they usually offer support. They provide support to the youths [living with HIV].* *(Participant 2, Male, 22 years old)*

Notes. # includes formal employment and self-employment

***** includes clinicians, nurses, HIV counsellors and community health workers
